# The out-of-the-delta hypothesis: dense human populations in low-lying river deltas served as agents for the evolution of a deadly pathogen

**DOI:** 10.3389/fmicb.2015.01120

**Published:** 2015-10-19

**Authors:** Yan Boucher, Fabini D. Orata, Munirul Alam

**Affiliations:** ^1^Department of Biological Sciences, University of Alberta, Edmonton, AB, Canada; ^2^Centre for Communicable Diseases, International Centre for Diarrhoeal Disease Research, Bangladesh (ICDDR,B), Dhaka, Bangladesh

**Keywords:** *Vibrio cholerae*, cholera, pandemic, epidemic, delta, pathogen, evolution, salt intrusion

## Abstract

Cholera is a diarrheal disease that has changed the history of mankind, devastating the world with seven pandemics from 1817 to the present day. Although there is little doubt in the causative agent of these pandemics being *Vibrio cholerae* of the O1 serogroup, where, when, and how this pathogen emerged is not well understood. *V. cholerae* is a ubiquitous coastal species that likely existed for tens of thousands of years. However, the evolution of a strain capable of causing a large-scale epidemic is likely more recent historically. Here, we propose that the unique human and physical geography of low-lying river deltas made it possible for an environmental bacterium to evolve into a deadly human pathogen. Such areas are often densely populated and salt intrusion in drinking water frequent. As *V. cholerae* is most abundant in brackish water, its favored environment, it is likely that coastal inhabitants would regularly ingest the bacterium and release it back in the environment. This creates a continuous selection pressure for *V. cholerae* to adapt to life in the human gut.

## Introduction

Bacteria that are pathogenic to humans need to be able to survive inside the human body, which is vastly different from any environment outside a eukaryotic host (Martínez, 2013). Many bacteria able to infect humans are adapted to other animal hosts, making the jump to survival in humans small evolutionarily. Such bacteria, which are transmitted from animals to humans, are called zoonoses. This is the case for *Yersinia pestis*, the causative agent of plague, of which there have been three pandemics, all originating from foci where this bacterium was found in rodent populations within areas densely populated by humans (Morelli et al., 2010). Other bacteria pathogenic to humans, such as *Pseudomonas aeruginosa*, are opportunistic and not necessarily associated with an animal vector, but rather found on the skin or in various environments such as air, water, and soil. These opportunistic pathogens are not specifically adapted to infect humans but can nonetheless survive inside the immune-compromised host (Martínez, 2014).

**FIGURE 1:**
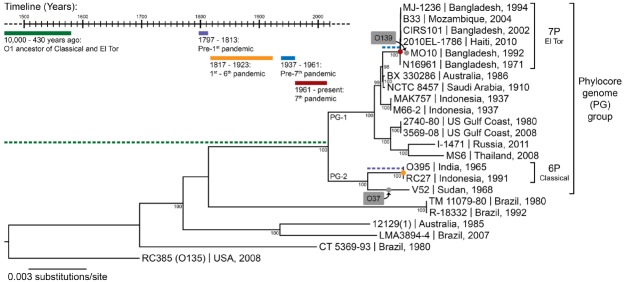
**The phylogeny of ***V. cholerae*** and history of cholera.** The maximum likelihood phylogenetic tree was constructed from a concatenated alignment of locally collinear blocks (2,602,418 bp) and using the GTR gamma substitution model with 100 bootstrap replicates (indicated on tree nodes). Non-O1 RC385 (O135) serves as the outgroup. The scale bar represents nucleotide substitutions per site. The geographical source and year of isolation are indicated next to the strain names. The gray boxes and dots indicate serogroup replacement by the ancestor of some strains of the phylocore genome (PG) group. The timeline shows significant events in the history of cholera, and their inferred positions in the phylogenetic tree are indicated by their corresponding color. 6P and 7P denote strains of the sixth and seventh pandemic, respectively.

Other pathogenic bacteria infecting humans are neither zoonotic nor opportunistic. *Vibrio cholerae*, the cause of the ancient disease cholera, is an environmental bacterium, living mainly in estuarine brackish water regions, either free-living or associated with the surface of various marine eukaryotes, such as algae, oysters, crustaceans, fish, or copepods (Colwell, 1996). *V. cholerae* can occasionally infect humans, even if they are healthy, and are therefore not opportunistic pathogens, but rather facultative pathogens, which vary in their pathogenic potential to humans from non-virulent to highly virulent (Haley et al., 2014). In the case of *Y. pestis*, the fact that warm-blooded animals (rodents) are its primary host can help explain its pathogenicity toward humans (Perry and Fetherston, 1997). For opportunistic pathogens such as *P. aeruginosa*, we understand that they do not have a specific adaptation to infecting humans but rather a general ability to do so when the host is compromised. For *V. cholerae*, however, it is not clear how a specifically coastal aquatic microbe would have evolved to become one of the deadliest pathogens in human history, killing millions through seven pandemics. *V. cholerae* is an extremely diverse species, harboring more than 200 different serogroups. Among all this diversity, only a single genetic lineage, the phylocore genome (PG) group, which is mostly composed of strains displaying the O1 serogroup, is responsible for all seven known pandemics (Figure 1). Did this transition to an ability to cause pandemics simply occur by chance or was it the result of ongoing natural selection?

## *V. cholerae* is Genetically Predisposed to Survive the Human Gut

Although most *V. cholerae* are not pathogenic to humans, any random strain of this species found in a coastal environment has several characteristics that would facilitate its survival in the human gut. The first is its ability to grow from 18 to 41°C, allowing it to proliferate in the human body. This is a rare trait among *Vibrio*, most species being unable to grow above 33°C (Brenner et al., 2005). Another key adaptation is the ability to survive a range of salinities, as it is mainly found in ocean to freshwater transition areas but can be isolated from waters with minimal amounts of salinity (Louis et al., 2003). This is also a rare trait among *Vibrio*, most of which tolerate a smaller range of salinities and have a higher minimal requirement for salt (Brenner et al., 2005). *V. cholerae* has to be able to survive low salt conditions to be ingested by humans drinking fresh or slightly brackish water.

*Vibrio cholerae* as a species also displays a number of other characteristics that likely result in a higher survival rate in the human gut. Members of this species naturally form biofilms in aquatic environments, often on the chitinous surface of various marine animals and zooplanktons (Meibom et al., 2004). Interestingly, this ability of *V. cholerae* to attach to chitin and form biofilms provides it with an indirect adaptation for survival in the human gut. The digestive tract contains abundant mucin, which is an analog of chitin. *V. cholerae* can degrade and bind to this mucin like it does to chitin (Wong et al., 2012). Therefore, the ability of *V. cholerae* to form biofilms on chitinous surfaces in aquatic environments likely facilitates this bacterium attachment and survival in the human gut mucilaginous surfaces. These biofilms have been shown to increase resistance to gastric acids and bile salts found in the intestinal tract, and also protect against immune responses (Hung et al., 2006; Almagro-Moreno et al., 2015; Teschler et al., 2015). *V. cholerae* is often shed from human hosts as biofilms, which enhances their infectivity and their survival in the environment. Indeed, while in biofilms, *V. cholerae* can be found in a hyperinfectious physiological state, and infectious dose for biofilm-derived *V. cholerae* can be orders of magnitude lower than that of free-living cells (Faruque et al., 2006). Biofilms also provide *V. cholerae* with resistance to eukaryotic grazing in the environment, enhancing their survival outside the host after shedding (Matz et al., 2005).

It has also been suggested that the type VI secretion system (T6SS), ubiquitously found in *V. cholerae*, could give it an edge in competing with the normal human gut flora (Fu et al., 2013). This system encodes a syringe-like structure that can pierce cellular envelopes of other bacteria and some eukaryotes, injecting effector proteins that can kill the recipient (Pukatzki and Provenzano, 2013; Unterweger et al., 2014). This could allow *V. cholerae* to kill competing bacteria native to the human gut, which are well adapted to this environment and could outcompete it. This secretion system likely evolved in nature because it allowed *V. cholerae* to compete more effectively with other bacteria or avoid predation from eukaryotes, as it has been shown to be effective against a range of microbes, including eukaryotes such as slime molds (Pukatzki et al., 2006).

Also important for bacteria pathogenic to humans is the regulation of gene expression. The expression of virulence factors only in a context in which they are useful (i.e., in the human gut) is critical to the long-term viability of a pathogen. *V. cholerae* displays quorum sensing control of numerous virulence factors, allowing it to produce toxins only when the population is sufficiently large to have an effect on the host (Zhu et al., 2002). Quorum sensing also controls biofilm formation, which we already mentioned as a virulence factor in *V. cholerae*. Another key part of gene regulation in *V. cholerae* is the ToxR regulon. The latter is a network of genes modulated by transcriptional regulators encoded by *toxRS*, *tcpPH*, and *toxT*. It modulates the expression of a number of virulence factors so that they are produced at the right time in the infection process. Although *tcpPH*, *toxS*, and *toxT* appear in low frequency in environmental *V. cholerae* strains, *toxR* is almost always found (Bina et al., 2003). Several other genes considered to be virulence factors are also very frequently found in *V. cholerae* strains regardless of their origin, including RTX (repeat in toxin), MSHA (mannose-sensitive hemolysin agglutination pilin), *pilE* (pilin), *hlyA* (hemolysin), and *nanH* (sialic acid degradation; O’Shea et al., 2004).

All these genetic traits, found in all or a majority of *V. cholerae* strains, almost undoubtedly evolved outside the context of a human host, as most members of this species are harmless residents of aquatic environments. These traits likely evolved by providing a survival advantage in marine coastal environments, such as protecting against predators, increasing resistance to various environmental conditions, outcompeting other bacteria, or associating with resource-rich zooplankton and phytoplankton (Martínez, 2013). For example, the T6SS is useful to defend against predation by eukaryotic grazers or competition with other microbes in the water column (Pukatzki et al., 2006). Various pilins can serve to adhere to surfaces and facilitate biofilm formation in aquatic environments (Almagro-Moreno et al., 2015). These traits provide a basic genetic background that fortuitously makes survival in a human host more likely, but not sufficient for *V. cholerae* to become a human pathogen.

Other virulence factors, found only in a minority of *V. cholerae* strains, add to this background and enhance their potential to cause various illnesses in humans, ranging from ear infection, to septicemia and gastroenteritis. One such factor is the type III secretion system (T3SS), which allows the delivery of effector proteins to a target cell (Chatterjee et al., 2009; Alam et al., 2011a). *V. cholerae* strains of various serogroups harboring the T3SS and/or some of the other virulence factors mentioned earlier have caused sporadic diarrheal episodes in Thailand (Dalsgaard et al., 1999), India (Mukhopadhyay et al., 1995), Peru (Dalsgaard et al., 1995), Nigeria (Marin et al., 2013), and numerous other countries (Dalsgaard et al., 2001; Chatterjee et al., 2009). These virulence factors alone, however, are not usually sufficient to cause an actual epidemic. Strains causing long-lasting and larger-scale outbreaks display two major virulence factors: the cholera toxin (CT) and toxin co-regulated pilus (TCP). Both of these are relatively rare in most environmental *V. cholerae* populations (O’Shea et al., 2004), but are found in all strains of the PG lineage responsible for pandemics (with a few exceptions, likely due to secondary loss; Chun et al., 2009). The CT is the main virulence factor of *V. cholerae*, causing massive release of electrolytes and water in the intestinal lumen through its activation of adenylate cyclase and consequent increase in intracellular levels of cyclic AMP (Vanden Broeck et al., 2007). The genes encoding this toxin are carried by a lysogenic phage, CTXΦ, inserted in the genome of *V. cholerae* (Waldor and Mekalanos, 1996; Faruque and Mekalanos, 2012). It has been shown that CTXΦ can be lost, gained *de novo*, or recombine if several phages are inserted in the genome of a single *V. cholerae* strain (Kim et al., 2015). This has led to a significant variation in the CT and associated genes of strains in the PG group (Safa et al., 2010). The selective advantage this phage provides or the nature of its association with PG *V. cholerae* strains in nature is such that it has remained almost ubiquitously present in members of this lineage, with occasional loss and regain episodes (Kim et al., 2015). TCP, the second major virulence factor of *V. cholerae*, is a pre-requisite to the presence of CTXΦ, as it is the receptor for that phage (Karaolis et al., 1999). TCP is also a virulence factor on its own, as it facilitates bacterial interaction through direct pilus-pilus contact required for microcolony formation, playing a role in adhesion and biofilm formation (Almagro-Moreno et al., 2015). The capacity to become toxigenic (produce CT) is believed to have evolved through the sequential acquisition of TCP and CTXΦ (Faruque and Mekalanos, 2003). Although the origin of these major virulence factors is unknown, homologs of both can be found in *Aliivibrio fischeri* symbionts of squid, a distant relative of *V. cholerae* (Ruby et al., 2005). The phage found in *A. fischeri* lacks the CT, but is otherwise very similar to CTXΦ. The TCP gene cluster of *A. fischeri* is missing a few genes present in its *V. cholerae* homolog, but is otherwise very similar. This suggests that other bacterial species could have been the source for the major virulence factors of *V. cholerae* from the PG group. The presence of CTXΦ and TCP is not limited to the PG lineage, and strains belonging to unrelated lineages with various serogroups have been found to contain one or both of these genes (Chun et al., 2009). Although these non-PG strains carrying CTXΦ and TCP have caused sporadic diarrheal episodes at various times across the world, the combination of virulence factors they harbor is different from that of PG strains and insufficient for causing actual pandemics of the scale observed historically for cholera. Of all *V. cholerae* strains that have caused outbreaks, only the PG group is known to have spread far and wide and survived through more than a few years as a lineage infecting humans. The main distinctive characteristics likely harbored by the ancestor of this group are the presence of CT, TCP (as part of the *Vibrio* pathogenicity island-1 or VPI-1), VPI-2, and the O1 antigen (Chun et al., 2009). A range of other factors contributing to pandemic potential is also likely present in this lineage, but the identity of many of them is still unknown. A study, which passaged a population of *V. cholerae* O1 transposon mutants through infant rabbits, found 133 genes contributing to survival in the mammalian host (Kamp et al., 2013). This included all the virulence factors already mentioned for PG *V. cholerae* O1, but also genes with identified functions in purine and pyrimidine biosynthesis and amino acid metabolism, as well as genes with putative function of phosphate acquisition, post-translational modification, fatty acid metabolism, and transport. This highlights that a constellation of genes was assembled to give pandemic potential to harmless aquatic *V. cholerae*. Lateral gene transfer is frequent among *V. cholerae* and most virulence factors are found on mobile elements such as pathogenicity islands, phages, or integrative conjugative elements (Faruque and Mekalanos, 2003). This means that various combinations of virulence genes must be regularly created in environmental reservoirs where *V. cholerae* strains interact. However, since the ability to become pandemic, as opposed to merely infecting humans or causing sporadic outbreaks, is a complex trait likely requiring over a 100 genes, sustained natural selection would be necessary for the incremental build-up of this complexity. What selective pressure made it possible for this combination of genes to be successfully fixed in a population of PG *V. cholerae* and maintained in most of its descendants for centuries? For bacteria linked to historically important epidemics or pandemics such as the plague, the general answer is relatively simple. Such zoonotic bacteria undergo selective pressure for adaptation to life in their warm-blooded animal hosts and are consequently able to survive in humans (Martínez, 2013). For example, plague pandemics resulted from the contact of rodent populations (*Y. pestis* primary host) with dense human populations in China (Morelli et al., 2010; Wagner et al., 2014). However, although *V. cholerae* can be found associated with zooplanktons such as copepods, these animals are invertebrates very different from the human host. This association is unlikely to be sufficient for the evolution of pandemic strains, and another source of long-term selection would be required.

## The History of Pandemic Cholera

How and where pandemic cholera first evolved is a fundamental question that has been elusive for decades. This is due to the difficulty of defining cholera precisely (as it has a broad clinical spectrum) and to distinguish it from other diseases that cause vomiting and diarrhea (Barua, 1992). Only since the advent of serological and molecular methods have we been able to diagnose cholera with reliability and trace the relationship between various epidemics.

Seven pandemics of cholera have been recorded in medical history, the first one starting in India in 1817 and the last in Indonesia in 1961 (Barua, 1992). Phylogenetic analyses based on whole genomes and multi-locus sequence typing suggest a common ancestor for strains of *V. cholerae* belonging to all seven pandemics (Figure 1). These pandemic strains and their close relatives have been termed the PG group (Chun et al., 2009), and their common ancestor has been estimated to date back to anywhere from the beginning of sedentary agriculture (10,000 years ago) to 430 years ago (Devault et al., 2014). There is little contention that cholera-like diseases were present in Asia and Europe since ancient times, based on descriptions of patient symptoms closely matching those of cholera found in ancient texts and records (Barua, 1992). The earliest texts describing cholera-like symptoms appeared in Greece (Hippocrates, fourth century B.C. and Aretaeus of Cappadocia, first century A.D.) and in India (Sushruta Samhita, fifth century B.C.). Reports of cholera from Arabic scholars Rhazes and Avicenna can be found in the tenth and eleventh centuries A.D. (Barua, 1992). However, these writings describe sporadic cases, not on the epidemic scale cholera is known for. *V. cholerae* strains outside the PG group can cause symptoms similar to pandemic cholera, and could have been the cause of these cases. Numerous reports of cholera can also be found for Europe between the sixteenth and eighteenth century. In Asia, the first written accounts in India after that of Sushruta Samhita started in 1503, shortly after the Portuguese settled in Goa, with some records for the disease being present in Delhi in 1325 and in Merwah in 1428 and again in Goa in 1563 (Barua, 1992). There were reports of the disease in India and neighboring countries such as Bangladesh and Indonesia throughout the seventeenth and eighteenth centuries. The frequent reports of cases matching closely the symptoms of cholera in Europe and Asia from the sixteenth century onwards, sometimes as epidemics, suggest that the disease was widespread before the first pandemic caused by the PG group of *V. cholerae* in 1817. However, *V. cholerae* was not isolated in pure culture until 1884 and historical medical records are hard to interpret, with frequent confusion of cholera with other diarrheal diseases before and even during the pandemics. It is therefore not possible to determine with certainty that these epidemics were caused by *V. cholerae* from the PG lineage.

There is evidence suggesting that pre-pandemic cholera cases were caused by strains genetically related to isolates from the pandemics. The PG group responsible for pandemics, includes only strains of the O1 serogroup (with a few exceptions that are clearly derived traits, such as the O139 serogroup in *V. cholerae* El Tor Bengal and O37 in *V. cholerae* V52 Sudan; Chun et al., 2009). It is divided into two main branches, which are significantly divergent from each other. One branch (PG-2) includes the classical biotype (responsible for the sixth and presumably the earlier pandemics) and the other (PG-1) includes the El Tor biotype (responsible for the current and seventh pandemic). Strains related to both the El Tor and classical biotypes but temporally or geographically unlinked to the pandemics are found in both PG-1 and PG-2 groups. The *V. cholerae* V52 strain isolated from a 1968 epidemic in Sudan, despite having a different serogroup, is clearly related to the ancestor of classical strains that caused the first six pandemics (Figure 1). Multiple strains related to the El Tor biotype, but not associated with the seventh pandemic, have also been found. These strains seem to be native to Australia (various rivers in Queensland), the USA (Gulf Coast), Thailand, and Russia, and are all of the O1 serogroup and clearly phylogenetically closely related to the seventh pandemic strains (Figure 1). This suggests that *V. cholerae* belonging to the PG-1 group had spread globally before the strain ancestral to the seventh pandemic emerged in Indonesia. The latter likely evolved locally, as epidemics caused by the El Tor biotype started in 1937 in Sulawesi (Indonesia), until they spread globally in 1961, starting the seventh pandemic (Salim et al., 2005). *V. cholerae* related to the ancestor of the classical strains that caused the first six pandemics are also likely to have been dispersed globally. The V52 Sudan strain was found far from the origin of the first pandemic in India, and is too divergent from sixth pandemic strains to have been descended from them, despite being part of the PG-2 group (Figure 1). Consequently, it is possible that the ancestor of the PG group had spread widely before the first pandemic, with its descendants locally evolving in India into the classical biotype and in Indonesia into the El Tor biotype (Feng et al., 2008). This would be consistent with cholera cases being widely observed in Europe and Asia well before the pandemics (Barua, 1992).

## A Proposed Origin of Pandemic Cholera in Historically Densely Populated River Deltas

We propose that extensive contact between *V. cholerae* and humans was made possible by high population densities in river deltas where humans drink the brackish surface waters where this bacterium lives. *V. cholerae* then circulates via the fecal-oral route in local populations and environmental reservoirs, leading to a selection and enrichment of variants capable of surviving in the human gut and eventually infecting individuals.

**FIGURE 2:**
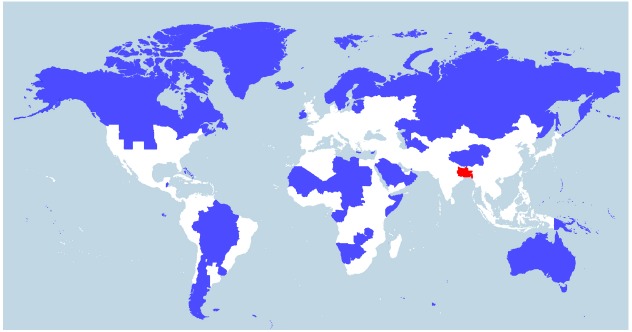
**World population density.** World map showing two different areas (blue and red) that each house 5% of the world population (394 million). Whereas the blue region encompasses several countries, the red region includes just one country—Bangladesh—and three eastern Indian states (Bihar, Jharkhand, and West Bengal) located in or near the Ganges Delta. The white region represents 90% of the world population. The map was constructed by Max Galka using the QGIS mapping tool with population data taken from the 2014 Credit Suisse Global Wealth Databook, used with permission (Galka, 2015).

Indeed, *V. cholerae* occurs widely in coastal areas as it strongly favors low salinities (between 2 and 14 ppt) and is tolerant of a wide range in temperatures (18–41°C; Louis et al., 2003; Brenner et al., 2005), with highest densities being achieved in warmer temperatures (>25°C; Takemura et al., 2014). People living in coastal areas of Bangladesh (the Ganges Delta), for example, ingest water from rivers or shallow wells with salinities averaging between 2.8 and 8.2 ppt and temperatures between 26 and 35°C during the dry season (Khan et al., 2011). Today, of the ∼400 million people inhabiting the Ganges River Basin (Figure 2), there are around 20 million living in coastal areas of Bangladesh who are affected by salt intrusion to varying degrees (Khan et al., 2011). If the proportion of the population in the Ganges Basin exposed to salt intrusion was historically similar to what is observed today, around 6–7 million people would have been regularly drinking brackish water in that area in 1800 A.D. (McEvedy and Jones, 1978). We suggest that such a large number of people constantly ingesting water at an ideal temperature and salinity for *V. cholerae* will be exposed to a significant amount of the bacteria. *V. cholerae* abundance can reach up to 6 × 10^3^ cells/ml in water (Heidelberg et al., 2002), or higher if they are found as biofilms on the surface or in the gut of zooplanktons, each microscopic animal carrying up to 1 × 10^4^ *V. cholerae* cells (Colwell, 1996). It is not possible to know the historical abundance of *V. cholerae* cells in waters of river deltas such as the Ganges, but it is unlikely that it would be much different from what is observed today in coastal areas across the world. Low-lying river deltas are the places more likely to put humans in contact with *V. cholerae*, as these are traditionally highly populated because of their fertile soils and their close contact with the ocean makes salt intrusion in drinking water likely. Deltas in warm regions, which have been densely populated for long historical periods, are therefore the most likely place for fecal-oral transmission and evolution of *V. cholerae* as a pandemic pathogen. Deltas outside Eurasia are less likely candidates, as their historical population densities are relatively lower (Goldewijk et al., 2010). Asia is home to many of the world’s largest and historically most densely populated deltas (the Indus in Pakistan, Ganges/Brahmaputra in India and Bangladesh, Mekong in Vietnam, Huang He and Yangtze in China) and is where all recorded cholera pandemics have started, making it the most likely region for the origin of this disease. The areas surrounding the Huang He, Yangtze, and Ganges rivers have been estimated as some of the most densely populated areas in the world from the beginning of the Holocene period (10,000 B.C. to the present; Goldewijk et al., 2010; Figure 2). The population of the Indian subcontinent was already estimated at over 30 million in 200 B.C., 20 million of which lived in the Ganges Basin (McEvedy and Jones, 1978). This population would grow to an estimated 200 million by the time the British took control around 1800 A.D., with an estimated 130 million in the Ganges Basin. China was similarly densely populated, with an estimated 40 million people in 200 B.C., mostly centered around the Huang He and Yangtze, growing to around 300 million in 1800 A.D. (McEvedy and Jones, 1978). These two areas alone represented 40% of the world population in 200 B.C. and over 55% in 1800 A.D. (McEvedy and Jones, 1978).

Although it is not possible to determine exactly from which of the world’s large river deltas cholera would have originated, the Ganges Delta is a likely candidate for several reasons. Among all of the world deltas, the only place where cholera has been continually endemic since the start of the pandemics is the Ganges Delta. Despite similar population densities and sanitary conditions, other deltas in Southeast Asia do not maintain cholera transmission, indicating that other local determinants are critical for the maintenance of cholera (Pascual et al., 2002). The Ganges Delta is unique in its pattern of cholera incidence. Most locations with endemic cholera have a single peak of cholera incidence during the warm season of the year, in which water temperatures rise. The Ganges Delta, however, has a dual peak pattern of cholera incidence, which is high in spring and fall. Low flow in the Ganges and Brahmaputra in the spring can help seawater intrusion, which is favorable to the growth of *V. cholerae*. The low flow also helps the movement of *V. cholerae* inland with marine phytoplankton and zooplankton to which it is associated (Akanda et al., 2009). Higher flow volumes of these rivers in monsoon season, which increases the extent of flood-affected areas, are responsible for high fall incidence (Bouma and Pascual, 2001). Cholera is more prevalent in coastal districts in the spring and in inland regions in the fall (Sack et al., 2003), suggesting that cholera enters the human population from infections initiated on the coast and then spreads inland, facilitated by shedding from infected individuals and spreading from high water levels and floods. Epidemics can also occur outside this regular pattern when abnormally high rainfall and associated flooding occurs (Alam et al., 2011b). This highlights that floodwaters transmit infectious clones of *V. cholerae*, which then circulates via the fecal-oral route, causing an epidemic.

There is also evidence of selective pressure exerted by cholera on the human population of the Ganges Delta. Blood group O is linked to an increased risk of severe cholera symptoms and has a low prevalence in the Ganges Delta area (Nelson et al., 2009). Other genetic factors are also likely to be involved in cholera susceptibility, as biologically related household contact of a patient are three times as likely to contract cholera as an unrelated contact living in the same household. Strong selective pressure has been detected on key pathways of innate immunity among the Bengali residents of Dhaka (Karlsson et al., 2013). Most people in Bangladesh have developed some immunity to cholera by the age of 10–15, vibriocidal antibodies being detectable in their blood.

Given this long-term influence of *V. cholerae* on human populations, it is likely that humans have also influenced the evolution of *V. cholerae*. The seventh pandemic has spread across the world in three waves since its start in 1961, each caused by a specific genetic variant. It has recently been shown that all three of these variants evolved in the Bay of Bengal region (Mutreja et al., 2011). The process by which established pandemic *V. cholerae* strains are successively globally replaced by novel variants is not well understood. Even if it is assumed that travelers, oceanic currents, and ship ballasts can disseminate novel strains worldwide (Ruiz et al., 2000), older established strains still have to be outcompeted by the newcomers. It has been suggested that existing human immunity to older variants in endemic areas could give an advantage to novel strains, but the possibility that environmental factors are involved in this evolution cannot be excluded (Kim et al., 2015). Consistent with the suggestion that humans have influenced the evolution of pandemic *V. cholerae* strains over the last two centuries, our hypothesis proposes that humans are also responsible for their origin. Through their constant ingestion and shedding of *V. cholerae* from environmental populations found in local brackish water sources, humans selected for variants capable of residing in their gut and causing cholera.

## Tracing the Origins of Pandemic Cholera

Our hypothesis implies several specific predictions, which could be falsified. It predicts that humans living in coastal areas and drinking brackish water where *V. cholerae* is present should harbor this bacterium in their gut. The residence time of *V. cholerae* in the gut of such brackish water consumers is an open question, but it is likely to be transient, as even pandemic strains rarely reside in the gut for long periods of time (Sack et al., 2004). A portion of strains found in the human gut should be specifically adapted to that environment and be a non-random subsample of the environmental population. There will also likely be an enrichment of such human-adapted genotypes in the environmental population, compared to coastal locations where no humans live. An analogy could be drawn here to *A. fischeri* symbionts of squids. These have specific genes allowing them to find the light organ of squids and survive there (Wier et al., 2010). They are released back in the environment every morning, where they mix with other *A. fischeri*, which are not adapted to colonizing squids.

Although non-pandemic (non-PG) *V. cholerae* have been found either alone or associated with the PG strains in various sporadic diarrhea episodes (Ramamurthy et al., 1993) or epidemics (Hasan et al., 2012), it as yet to be found in the gut of healthy individuals. This is because our focus during epidemics is naturally on patients, and microbes are usually isolated from individuals that are sick, not the water that they drink or food they consume. This results in vast amount of information on the pandemic *V. cholerae* strains, but very little on other strains that could also be present in the gut of healthy humans. Culture-based studies, although sometimes done on samples from household contacts of cholera patients who could be healthy, usually target PG *V. cholerae* specifically (by using antibiotic selection and enrichment cultures) and rarely allow the identification of other strains. Culture-free studies of the human gut suffer from the same sampling bias toward infected patients. Although the gut flora of numerous healthy humans has been analyzed through microbiome studies across the world, we have yet to sample healthy humans regularly drinking coastal brackish water. Furthermore, current molecular analyses of the human gut is limited to the 16S *rRNA* gene, which lacks the resolution to differentiate between various *Vibrio* species that could be found there (Hsiao et al., 2014). It is of interest that one study using the 16S *rRNA* gene to look at the gut microbiota of children with cholera found traces of *Vibrio* a month after patients were admitted, even though the infection was treated successfully in a few days (Monira et al., 2013). This raises the possibility that *V. cholerae* could reside in the gut for prolonged periods of time. It is also known that pandemic *V. cholerae* infections can often be asymptomatic, meaning that the bacteria can be present in the human gut without being detected (King et al., 2008).

A second prediction of our hypothesis is that virulence factors contributing to cholera, such as CT and TCP, should be more abundant in an environment selecting for such factors. There should also be a larger diversity of virulence determinants. For example, a comparison of such genes in the Ganges Delta and a coastal cholera-free location in North America should reveal a higher abundance and variety of virulence factors in the former than the latter. This would be due to an enrichment of those factors in the general *V. cholerae* population because of ongoing selection in the human gut.

If the origin of pandemic PG group *V. cholerae* is not very ancient, it could also be possible to trace it to a particular geographical area. Extent relatives of the PG lineage could be found in a specific delta, similarly to what has been done to trace the origins of plague pandemics inside or near China (Morelli et al., 2010). Biogeographical study of the human pathogen *Vibrio parahaemolyticus* (mostly seafood-borne) shows that it is not panmictic (with all individuals free to move between locations), but that the oceans surrounding Asia have a distinct gene pool (Cui et al., 2015). Since *V. parahaemolyticus* is a marine organism, as opposed to *V. cholerae* which is mostly estuarine, the latter is likely to show even more biogeographical structure in its global distribution. If extent relatives of the PG lineage can be found, it is likely that its geographical origin could be confirmed. This would likely require extensive sampling of *V. cholerae* across the world, but such an effort is not beyond what has been done for several other bacterial pathogens.

It has recently been proposed that dysbiosis, a change in the proportion of various species in the normal microbiota, promotes lateral gene transfer (Stecher et al., 2013). *V. cholerae* infections often cause dysbiosis, as a massive bloom of *V. cholerae* in the intestine completely changes the normal microbiota (Hsiao et al., 2014). In a scenario in which *V. cholerae* progressively adapts to the human gut by regular ingestion, lateral gene transfer from gut microbes is likely, and traces of such transfer might be found inside its genome.

## Concluding Remarks

*Vibrio cholerae* is unusual as a human pathogen, as some representatives of this species can cause pandemics but are not zoonotic bacteria, instead having an environmental reservoir in coastal waters. This makes the evolution of variants with epidemic potential a fascinating mystery. A single phylogenetic lineage is known to have led to all seven pandemics, making evolution of pandemic variants a rare event. As for other human pathogens, extended contact with our species or other warm-blooded animals is likely necessary to yield highly virulent lineages. For a coastal bacterium, such contact was possible in human populations regularly drinking warm brackish waters, the favored environment of *V. cholerae*. We could be able to determine where pandemic cholera originated through a biogeographical study of the *V. cholerae* species, if the PG lineage is not panmictic. It might also be possible to understand the process through which it evolved by investigating the gut microbiota of healthy humans consuming water affected by salt intrusion from the ocean. This would represent a local impact of human population on a bacterial species, influencing its evolution with dire consequences.

## Author Contributions

YB wrote the manuscript, FDO revised the manuscript and created the figure, MA contributed the original hypothesis idea and revised the manuscript.

### Conflict of Interest Statement

The authors declare that the research was conducted in the absence of any commercial or financial relationships that could be construed as a potential conflict of interest.
